# Vascular Damage in Patients with Nonalcoholic Fatty Liver Disease: Possible Role of Iron and Ferritin

**DOI:** 10.3390/ijms17050675

**Published:** 2016-05-05

**Authors:** Giuseppina Pisano, Rosa Lombardi, Anna Ludovica Fracanzani

**Affiliations:** Department of Pathophysiology and Transplantation, Ca’ Granda IRCCS Foundation, Policlinico Hospital, University of Milan, Centre of the Study of Metabolic and Liver Diseases, Via Francesco Sforza 35, 20122 Milan, Italy; pinaz81@hotmail.com (G.P.); rosalombardi@hotmail.it (R.L.)

**Keywords:** NAFLD, ferritin, iron, cardiovascular disease, metabolic syndrome

## Abstract

Non Alcoholic Fatty Liver Disease (NAFLD) is the most common chronic liver disease in Western countries. Recent data indicated that NAFLD is a risk factor by itself contributing to the development of cardiovascular disease independently of classical known risk factors. Hyperferritinemia and mild increased iron stores are frequently observed in patients with NAFLD and several mechanisms have been proposed to explain the role of iron, through oxidative stress and interaction with insulin metabolism, in the development of vascular damage. Moreover, iron depletion has been shown to decrease atherogenesis in experimental models and in humans. This review presents the recent evidence on epidemiology, pathogenesis, and the possible explanation of the role of iron and ferritin in the development of cardiovascular damage in patients with NAFLD, and discusses the possible interplay between metabolic disorders associated with NAFLD and iron in the development of cardiovascular disease.

## 1. Introduction

Non Alcoholic Fatty Liver Disease (NAFLD), the most common chronic liver disease in Western countries, was previously indicated as the hepatic expression of the metabolic syndrome (MetS) having shared many similar clinical manifestations [1]. More recently it has been proposed that NAFLD precedes the development of type 2 diabetes and metabolic syndrome [2], significantly increasing the risk of incident type 2 diabetes [3] even in non-overweight subjects [4]. Recent evidence links NAFLD to increases of cardiovascular risk, and further studies reveal that the first causes of death in NAFLD patients are cardiovascular disease (CVD) [5,6,7,8] and cancer [5,9,10,11], and not just liver diseases. NAFLD is also considered by recent studies to be a risk factor in itself to the development of CVD independently of classical known risk factors [12]. Increased ferritin and body iron stores are frequently observed in patients with NAFLD [13,14]. Iron, through oxidative stress and interaction with insulin metabolism [15], can promote the development of vascular damage. Moreover, iron depletion has been reported to decrease atherogenesis in experimental models and in humans [16,17].

## 2. Ferritin, Insulin Resistance, Metabolic Syndrome, and NAFLD

Growing evidence proposes a correlation between serum ferritin, insulin resistance, and NAFLD [18,19]. Several studies reported a link between high ferritin levels and MetS [20], and its single components [21], with a linear increase with the increasing number of MetS components [20]. Liver fat accumulation is considered to be one of the first pieces of evidence in the development of insulin resistance, and a strong association between NAFLD, insulin resistance, and MetS features has been demonstrated [19,22,23]. The association between ferritin and components of the MetS has been suggested to be related to an undiagnosed NAFLD. Zelber-Sagi *et al.* [24] demonstrated that insulin was the strongest predictor of increased serum ferritin levels and, *vice versa*, ferritin has been proposed as a marker of insulin resistance [25].

The evidence that increased ferritin levels precede the development of diabetes was demonstrated in prospective studies [26,27], however, it is not well defined if increased ferritin (expression of body iron accumulation) could induce metabolic alteration. In chronic liver disease hyperferritinemia may be caused by an augmented release of the protein from injured hepatocytes. Pro-inflammatory cytokines, in fact, stimulate the synthesis of ferritin, which is an acute phase reactant [28]. In patients with NAFLD (in whom ferritin and body iron are frequently increased [13,29]) inflammation, metabolic alterations, and hepatocytes necrosis may coexist with a mild iron overload, all leading to hyperferritinemia [30,31]. In addition, even a small amount of hepatic iron accumulation combined with other cofactors can increase oxidative stress responsible for liver cell necrosis, activation of hepatic stellate cells, and fibrosis [19,32], implying that iron could also play a role in the progression from “benign” fatty liver to non-alcoholic steatohepatitis (NASH). The same mechanisms determining liver damage might act in the vessel walls.

Epidemiological studies indicated that ferritin not only is a marker of insulin resistance but also is one of the strongest risk factors for the progression of carotid atherosclerosis [33,34]. Confirming this observation, the removal of iron by phlebotomy was found to improve insulin resistance, liver function tests [13,35], and atherosclerosis [36]; however, mainly due to the small sample size of the studies, the impact of phlebotomy in NAFLD is still debated [37].

## 3. Iron and Atherosclerosis

The role of iron in the development and progression of atherosclerosis has been reported in several papers. Iron deposition, especially in macrophages of arterial walls, is increased in atherosclerotic lesions [14,38], and has been proposed as a marker of cardiovascular risk [16]. The role that iron plays in atherosclerosis has been hypothesized to be an increase in vascular oxidative stress and acceleration of arterial thrombosis [39]; this could be caused by the induction of oxidative stress catalysis, promotion of insulin resistance [15], decreased plasma antioxidant activity, increased low-density lipoprotein (LDL) oxidation [40], and enhanced macrophage activation determining oxidized LDL uptake [41].

Iron depletion in experimental models has been shown to decrease atherogenesis [17], while, in humans, blood donation has been associated with decreased risk for myocardial infarction [26], and phlebotomy has been suggested to decrease the progression of peripheral vascular disease [42].

A worse cognitive performance in patients with metabolic alterations—as a potential consequence of vascular damage, or directly as a neurodegenerative alteration—has been described in relation to iron status in animal models, and more recently in humans as well [43]. In insulin resistant obese patients a worse cognitive performance was found related with brain iron load in the caudate, lenticular nucleus, hypothalamus, and hippocampus (by magnetic resonance imaging (RMI)) and with increased hepatic iron concentration. It is possible to hypothesize that in presence of insulin resistance, the excess of iron, being highly reactive and promoting the generation of hydroxyl radicals, may cause both metabolic distress in the liver and alterations in some target brain areas [44].

## 4. Iron and Carotid Plaques: Arterial Iron Promotes Plaque Instability

Through the use of electron paramagnetic resonance spectroscopy Stadler *et al.* [45] were able to quantify iron in *ex vivo* carotid lesions and in healthy human arteries and, in doing so, found that iron in the carotid lesions was higher than in healthy subjects. They also found a correlation between cholesterols and iron accumulation in the lesions.

Lapenna *et al.* [14] in studying *ex vivo* carotid endo-arterectomy specimens found a significant correlation between serum ferritin and low molecular weight iron. Yuan *et al.* and Li *et al.* [46,47] suggested that iron found in atherosclerotic vascular tissue, generated mostly by erythrophagocytosis, could interact with lipoproteins in macrophages and be responsible for increased oxidative stress and their transformation into foam cells in the presence of an atherogenic environment. Thus the increase of iron in macrophages might contribute to vulnerability of human atheroma. Moreover, Li *et al.* reported, in *ex vivo* human carotid atherosclerotic lesions [48], the positive correlation of transferrin receptor 1 (TfR1) expression and macrophage infiltration, ectopic lysosomal cathepsin L, and ferritin expression and they suggested that the expression of TfR1 and ferritin in CD68 positive macrophages was correlated with the severity of human carotid plaques.

## 5. Ferritin and Atherosclerosis

Ferritin is considered a marker of atherosclerosis progression [33] and a relationship has been proposed between its levels and carotid atherosclerosis [34] in epidemiological studies. Moreover, ferritin was found associated with carotid intima-media thickness (IMT), and with the presence of carotid plaques in a large cohort of NAFLD patients [49]. In this paper the authors described a stronger association of ferritin with plaques rather than with increased IMT, hypothesizing that iron, by favoring endothelial damage and thrombosis [39], can promote the development of atherosclerotic complications. In NAFLD ferritin can reflect oxidative stress, inflammation, and hepatic necrosis. This protein has been found strongly associated not only with parameters influencing iron stores, such as sex, age, alcohol, and genetic factors *(i.e.*, *HFE* mutations), but also with metabolic alterations defining the metabolic syndrome. However, a correlation was described between ferritin and vascular damage that was independent from factors associated with metabolic syndrome [50,51,52].

These data were recently confirmed in a Chinese population study in which serum ferritin was found significantly increased in patients with abnormal glucose metabolism and related with IMT progression [53].

## 6. *HFE* Gene Mutations in NAFLD and Atherosclerosis

Several studies analyzed the role of HFE mutations in patients with NAFLD and iron overload. Valenti *et al.* [29] demonstrated that carriers of the C282Y mutation have lower insulin release and develop NAFLD in the presence of less severe metabolic abnormalities. This suggests that heterozygosis for the HFE mutation (responsible for mild iron overload) may trigger the clinical NAFLD manifestation [29]. More controversial is the role of HFE mutations in the development of atherosclerotic damage. In fact, while the atherogenetic role of iron has been reported (as observed in macrophages of arterial walls in atherosclerotic lesions [40,41] and in the beneficial effect of iron depletion on vascular damage [17], a lack of association between HFE mutations with vascular damage has been reported [54]. A faster clearance of iron from arterial lesions could be caused by a decrease of Hepcidin, which could facilitate iron export from macrophages [49].

## 7. Hepcidin, Macrophage Iron, and Vascular Damage

**Figure 1:**
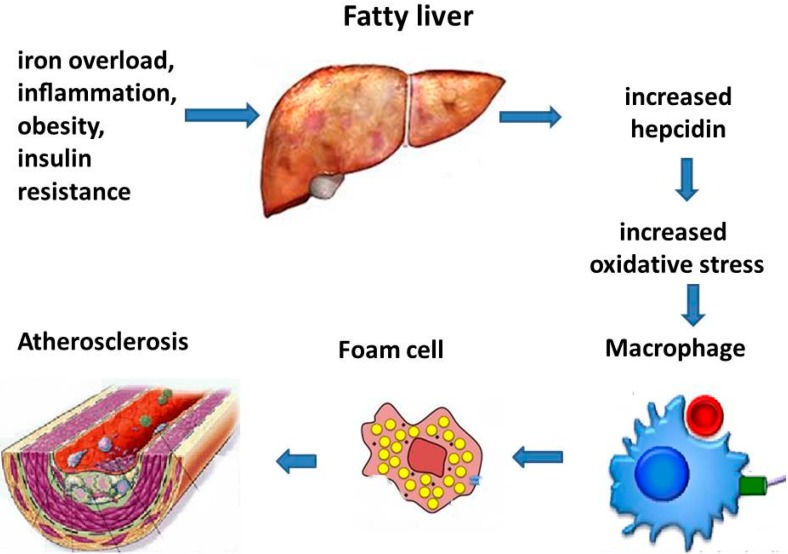
Simplified pathophysiological mechanisms of iron induced vascular damage through fatty liver.

Hepcidin, mainly produced in the liver, is defined as the key hormone regulating iron balance [55]. Hepcidin provides a defense mechanism against pathogens during inflammation by inhibiting iron recycling from macrophages and iron absorption from enterocytes. Also, in patients with metabolic disease, such as NAFLD, the deregulation of hepcidin expression/activity contributes to increased iron stores [56]. Subclinical inflammation and obesity can induce Hepcidin [57] and cause iron trapping in macrophages [58] in the presence of an atherogenic environment. Excessive iron in macrophages could be responsible for increased oxidative stress and transformation into foam cells. Sullivan *et al.* [16] suggested that increased hepcidin may generate iron induced atherogenesis and cardiovascular damages (Figure 1).

### Experimental Models

Findings from animal models of atherosclerosis and from studies of human atherosclerotic plaques provide evidence that elevated arterial iron levels may cause atherosclerosis. Both animal studies and clinical evidence indicate that in the presence of iron deficiency (*i.e.*, anemia) that iron can be mobilized from arterial plaques to be used in erythropoiesis with consequent iron reduction in the plaques.

Valenti *et al.* [59] reported the effect of the manipulation of intracellular iron on the release of atherogenic cytokines in human differentiating monocytes of patients with NAFLD, with Metabolic Syndrome, and with mild iron overload by treatment cells with iron salts or with hepcidin. Macrophages, but also the smooth muscle and the endothelial cells treated with iron salts, increased the release of the macrophage chemo attractant protein (MCP-1), an atherogenic chemokine that plays an important role in both the initiation and progression of atherosclerosis. Moreover, the iron salt treatment increased the IL-6 a proinflammatory cytokine involved in the acute phase response, independently of oxidative stress. IL-6 serum levels have been reported to correlate with vascular risk and with the inflammation within atherosclerotic plaques [60]. In addition it has been found that higher MCP-1 represents a negative prognostic factor in acute coronary syndromes [61]. The effect of hepcidin on MCP-1 release was similar to that of iron salts as it blocked cellular iron export. Furthermore, in patients with NAFLD and MetS, the iron-dependent induction of MCP-1 and IL-6 was found associated with the severity of vascular damage as it promoted macrophage activation by iron and may be involved in the pathogenesis of vascular damage progression. These results have also been observed in monocytes of healthy subjects in which iron treatment determined the induction of MCP-1 transcription and release, suggesting that this depicted a physiological response to increased intracellular iron availability [49].

## 8. Iron Depletion and Atherosclerosis

It has been reported that iron depletion decreases atherogenesis in experimental models [17]. In addition, iron reduction by frequent blood donations was found to be associated with decreased intima-media thickness [36] and decreased risk of myocardial infarction [26]. Thus, iron reduction potentially offers a benefit in atherosclerotic vascular disease acting as an anti-inflammatory process. However, the role of blood donation on cardiovascular diseases is not yet defined. The Nebraska Diet Heart Study [62], has established a relationship between blood donation and risk of cardiovascular events. This study evaluated the cardiovascular events in 655 individuals who had donated at least one unit of blood in the preceding 10 years and in 3200 who had not. The results indicated that, compared to non-donors, the blood donors showed a significant reduction of events such as myocardial infarction, angina, or stroke. They also had fewer cardiovascular procedures and less use of nitroglycerin. Nevertheless, it is not possible to rule out that blood donors have less cardiovascular events in connection to them being in apparently good enough health to be eligible to donate blood. The beneficial effect of blood donations on cardiovascular disease has been debated in a number of epidemiological studies [13,35,36,37]. Interestingly, Zacharski *et al.* [42], in a multicenter prospective trial conducted in veteran participants with peripheral arterial disease, showed that the beneficial effect of phlebotomy was present only in younger patients. This suggests that levels of body iron stores might be operative in the early phase of atherosclerosis, while hypercoagulability and diabetes mellitus in later-stages of the diseases. Low body iron may protect against atherosclerotic CVD through different ways: (1) limiting oxidation of LDL cholesterol [63]; (2) decreasing the clinical activity of myeloperoxidase [64]; (3) increasing high density lipoprotein (HDL) and apolipoprotein A (ApoA) [65]; (4) improving nitric-oxide mediated, endothelium-dependent vasodilation [66], and, finally, improving insulin sensitivity [67].

In addition, iron depletion has been demonstrated to improve insulin resistance [13] in NAFLD, while more controversial is the beneficial effect on liver histology in NASH [68,69]. About one third of patients with NAFLD and MetS have been reported to have dysmetabolic iron overload syndrome [70], and both venesection therapy (in the absence of weight loss) and dietary treatment have been shown to improve ferritin, metabolic parameters, and liver enzymes [70,71].

An imbalance of the homoeostatic mechanisms—including the interaction of iron with hepcidin, ferritin, insulin, and with adipokines and pro-inflammatory molecules—causes parenchymal siderosis that contributes to organ damage such as pancreatic β-cell dysfunction, liver fibrosis, and atherosclerotic plaque growth and instability. *Vice versa*, iron depletion could exert beneficial effects, not only in NAFLD patients with mild iron overload but also in healthy frequent blood donors [72].

## 9. Dietary Iron, Microbiota, and CVD

Elements such as dietary macronutrients, particularly the types of fats and carbohydrates, are known factors in the etiology of type 2 diabetes, a metabolic disease closely related with NAFLD, while more controversial is the effect of dietary iron. Iron is a transitional metal, strong pro-oxidant, and catalyzer of several cellular reactions that result in the production of reactive oxygen species, thereby consequently increasing the level of oxidative stress. Graham *et al.* [73] reported an increase in liver cholesterol biosynthesis in mice caused by high dietary iron, showing how iron could influence cholesterol levels and cause the development of fatty liver disease. In addition, the high dietary cholesterol promotes the development of fatty liver in guinea pigs which in turn leads to the dysregulation of iron metabolism because of damaged liver [74]. Iron dextran increased oxidative stress, which was associated with the altered expression of genes related to lipid metabolism and therefore contributing to hyperlipidemia [75]. The observations, obtained in animal models, that iron can modulate lipid metabolism and therefore be associated with liver and vascular damage are very promising but not yet consolidated in humans. Also, the effect of dietary iron is not well established [76] in humans, although the intake of heme iron before and during pregnancy has been reported to correlate with the onset of diabetes, a well-known risk factor for CVD [77]. Interestingly, iron deficiency also has been reported to be associated with increased CVD risk. Iron deficiency is associated with thrombocytosis due to the lack of inhibition of thrombopoiesis with consequent increases of thrombotic complications as reported in iron-deficient children and adults [78]. In addition iron deficiency (causing anemia) increases the risk of heart failure by causing tissue ischemia with consequent increased oxidative stress, which could damage myocardial cells [79].

**Figure 2:**
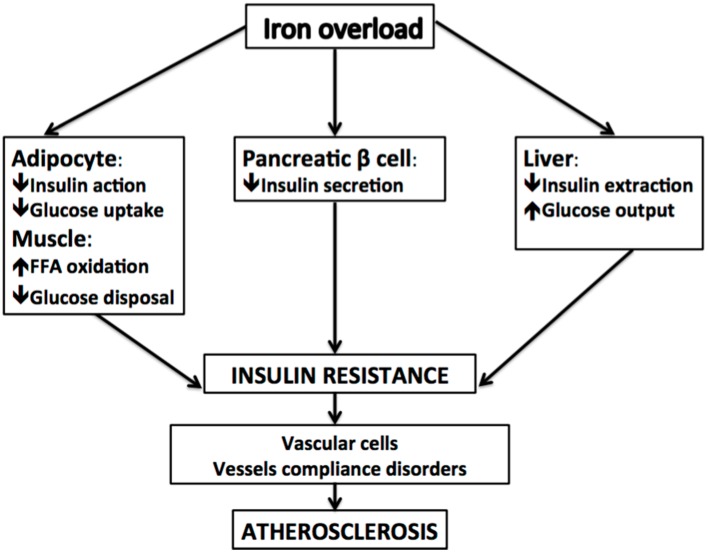
Effect of dietary iron overload on metabolic alterations, insulin resistance, and atherosclerosis. The downward arrows mean decrease and upward arrows mean increase. FFA: free fatty acid.

An updated review of cross-sectional, longitudinal, and intervention studies [79] evaluating the relation between iron and cardiovascular risk indicated that concentrations of iron within normal ranges does not have dangerous effects. In contrast, elevated amounts of non-protein-bound iron (free Fe), which has been reported to increase circulating homocysteine [80,81,82], seems to play a role in atherosclerosis. Free Fe catalyzes the formation of oxygen free radicals and oxidized low-density lipoprotein, which are well-established risk factors for vascular damage, thereby supporting the hypothesis that circulating homocysteine could be in part a surrogate marker for free Fe [83]. However, different iron types might act differently on the cardiovascular risk. Higher dietary intake of heme iron was found to be associated with increased cardiovascular risk; this association was not observed with non-heme and total iron intake [84]. De Oliveira Otto *et al.* [85] in a population study analyzing diet micronutrients indicated that dietary intake of non-heme iron was inversely associated with homocysteine, whereas high red meat intake (a predominant source of heme iron) was found to be associated with C-reactive protein. In addition, it is possible that the intake of nutrients containing non-heme iron (which is found in vegetables, cereals, and fruits) is more common in individuals with a healthy lifestyle (e.g., non-smokers and physically active individuals), while heme iron (abundant in red meat), which was found to be associated with insulin resistance, increased oxidative stress and CVD (Figure 2). In addition, red meat is also rich in choline and carnitine, both processed by enteric microbiota, and found to be related with atherosclerosis [86,87]. Dose-response analyses revealed a 7% increase in the risk of cardiovascular disease for each 1 mg/day increase in dietary heme iron [84].

A clinically important association between bacterial infection and CVD has been reported [88]. One of the possible mechanisms in the pathogenesis of atherosclerosis could be represented by the host immunological response of extravascular tissues and/or vascular walls to bacterial agents. It is known that gut microbiota may interfere with the host metabolism by promoting multiple functions, from development of the intestinal immune system to hepatic and energy metabolism. More recently it has been reported that specific forms of gut microbiota are present in the blood of patients with diabetes and atherosclerotic plaques, thus gut microbiota could represent an environmental risk factor for CVD [89]. Gut microbiota could have a direct proatherogenic influence in atherosclerosis plaque colonization through the bloodstream after events that affect the gut barrier. Both aberrant microbiota profiles and the flux of metabolites derived from gut microbial metabolism of choline, phosphatidylcholine, and L-carnitine have been found to be associated with metabolic disease, and contribute directly to cardiovascular diseases. However, although recent data on the role of microbiota in the development of NAFLD and progression to NASH are promising, particularly in animal models, conclusive results in humans on the effect of microbiota are still missing. Oral iron intake or food rich in heme iron could alter gut microbial composition and function providing one explanation for increased vascular disease risk [90].

## 10. Conclusions

In patients with NAFLD, hyperferritin and mild increases in body iron store are frequently detected and associated with vascular damage. Different mechanisms have been proposed to explain the atherogenic role of iron leading to increases in vascular oxidative stress and the acceleration of arterial thrombosis. Inflammation, metabolic alterations, and hepatocytes necrosis may coexist with a mild iron overload, all leading to hyperferritinemia, which is considered to be an independent predictor of cardiovascular damage. Iron depletion, achieved by phlebotomy, has been reported to improve insulin resistance and to reduce cardiovascular risk and damage. Finally, dietary strategies, which modulate the gut microbiota and different metabolic activities, could represent efficacious tools for reducing cardiovascular risk.
